# Adaptive evolution of complex innovations through stepwise metabolic niche expansion

**DOI:** 10.1038/ncomms11607

**Published:** 2016-05-20

**Authors:** Balázs Szappanos, Jonathan Fritzemeier, Bálint Csörgő, Viktória Lázár, Xiaowen Lu, Gergely Fekete, Balázs Bálint, Róbert Herczeg, István Nagy, Richard A. Notebaart, Martin J. Lercher, Csaba Pál, Balázs Papp

**Affiliations:** 1 Synthetic and Systems Biology Unit, Institute of Biochemistry, Biological Research Centre of the Hungarian Academy of Sciences, Temesvári krt. 62, Szeged H-6726, Hungary; 2 Department for Computer Science, Heinrich Heine University, Universitätsstraße 1, Düsseldorf D-40221, Germany; 3 Department of Bioinformatics (CMBI), Radboud University Medical Centre, Geert Grooteplein Zuid 26–28, Nijmegen 6525 GA, The Netherlands; 4 SeqOmics Biotechnology Ltd, Vállalkozók útja 7, Mórahalom H-6782, Hungary; 5 Sequencing Platform, Institute of Biochemistry, Biological Research Centre of the Hungarian Academy of Sciences, Temesvári krt. 62, Szeged H-6726, Hungary; 6 Department of Internal Medicine, Radboud University Medical Center, Geert Grooteplein Zuid 8, Nijmegen 6525 GA, The Netherlands

## Abstract

A central challenge in evolutionary biology concerns the mechanisms by which complex metabolic innovations requiring multiple mutations arise. Here, we propose that metabolic innovations accessible through the addition of a single reaction serve as stepping stones towards the later establishment of complex metabolic features in another environment. We demonstrate the feasibility of this hypothesis through three complementary analyses. First, using genome-scale metabolic modelling, we show that complex metabolic innovations in *Escherichia coli* can arise via changing nutrient conditions. Second, using phylogenetic approaches, we demonstrate that the acquisition patterns of complex metabolic pathways during the evolutionary history of bacterial genomes support the hypothesis. Third, we show how adaptation of laboratory populations of *E. coli* to one carbon source facilitates the later adaptation to another carbon source. Our work demonstrates how complex innovations can evolve through series of adaptive steps without the need to invoke non-adaptive processes.

## Abstract (web summary)

A fundamental question in evolutionary biology is how complex innovations requiring multiple genetic changes arise. Here the authors provide lines of evidence that changing environments facilitate the adaptive evolution of complex metabolic innovations via stepwise acquisition of single reactions.

## Graphical abstract

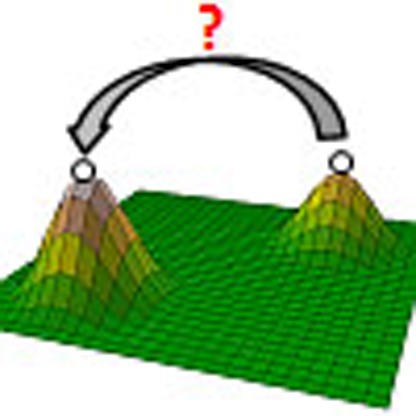

Evolutionary novelties frequently depend on the fixation of multiple, highly specific mutations, where intermediate stages of evolution seemingly provide little or no benefit^1^. Such complex adaptations are widespread in molecular networks and include the origin of multimeric protein machineries, establishment of interactions between transcription factors and their binding sites, receptor–ligand interactions and multi-step metabolic pathways^2^,^3^,^4^. According to the notion that evolutionary adaptation proceeds by the sequential fixation of single beneficial mutations^5^, complex adaptations are expected to occur only sporadically. One theory suggests that many evolutionary innovations, that is, qualitatively new adaptive traits, have non-adaptive origins, where neutral mutations prepare the ground for later beneficial mutations that lead to innovations^6^,^7^. Evidence for this process comes from laboratory evolution of RNA enzymes^8^, but its role in the establishment of complex molecular pathways remains unclear. In the case of metabolic networks, the theory proposes that ‘many additions of individual reactions to a metabolic network will not change a metabolic phenotype until a second added reaction connects the first reaction to an already existing metabolic pathway'^7^. However, this non-adaptive process is expected to be extremely slow, and furthermore, there is no direct empirical support for this scenario in bacteria, which are especially prolific in producing metabolic innovations. Although free-living bacteria increase their genome size through horizontal gene transfer and gene duplication, their genomes remain compact, and non-functional sequences appear to be rare compared with most eukaryotes^9^. Genes under relaxed selection are rapidly inactivated and subsequently lost in free-living bacteria, not least because there is a pervasive mutational bias towards deletions of genomic segments^9^. Consequently, genes encoding functionally completely intact enzymes that provide no immediate fitness advantage are generally unlikely to be maintained for long periods. Even under a scenario where the neutral intermediate-step mutation is not required to reach high population frequencies (that is, ‘stochastic tunnelling'^10^), evolution is expected to be slower than traversing purely adaptive trajectories through natural selection. Thus, understanding the evolution of complex innovations remains a formidable challenge.

Previous population genetic models^11^ and computer simulations of genetic circuits and RNA molecules^12^ offer a potential solution to the problem of complex adaptations. These works indicate that complex or temporally fluctuating conditions can facilitate adaptation, partly by allowing populations to escape fitness plateaus and reach new adaptive peaks. Similarly, a study on digital organisms revealed that populations often evolve complex features by building on simpler functions that had evolved earlier^13^. However, the extent to which these abstract considerations apply to specific cellular subsystems remained unknown, partly due to the shortage of systems-level analysis that would combine computational modelling and evolutionary experiments.

In this work, we focus on bacterial metabolic networks to examine how novel nutrient utilization phenotypes can be acquired via the addition of new reactions to an organism's enzyme repertoire. While not all complexity at the level of molecular systems are expected to provide a functional advantage^14^,^15^, metabolic pathways utilizing novel nutrients arguably qualify as adaptive traits. The problem of the evolution of novel metabolic pathways has two complementary aspects, relating to their origin and subsequent evolutionary establishment across multiple species. Previous works were largely concerned by how novel biochemical reactions arise first during the course of evolution^16^,^17^. In this paper, we ask how existing enzymatic reactions can assemble to form a novel metabolic pathway in an organism that already harbours a complex metabolic network. We extend and generalize an early suggestion that varying nutrient environments play a prominent role in the establishment of biosynthetic pathways^16^.

Specifically, we employ detailed simulations on a pan-genome scale to demonstrate that complex metabolic innovations can evolve via the successive acquisition of single biochemical reactions that each confers a benefit to utilize specific nutrients. Thus, temporal changes in nutrient availability or complex environments (where multiple nutrients are available) can facilitate adaptive evolution of metabolic pathways through the step-by-step expansion of metabolic niches. Gene acquisition patterns across bacterial genomes and *de novo* laboratory evolution of nutrient utilizations in *Escherichia coli (E. coli)* provide clear support for the hypothesis.

## Results

### Most metabolic innovations demand only a few novel reactions

In this work, we systematically studied the expansion of metabolic networks. We specifically asked whether metabolic innovations can evolve in a purely Darwinian manner through series of adaptive steps. Our starting point was the previously reconstructed metabolic network of *E. coli* K-12, arguably the best studied and most reliable reconstruction of a genome-scale metabolic system, composed of 2,077 unique reactions, including transport processes^18^. Previous studies showed that bacterial networks expand largely by acquiring genes involved in the transport and catalysis of external nutrients, driven by adaptations to changing environments^19^. On the basis of these observations, here we studied the potential selective advantages conferred by the addition of new metabolic reactions to the *E. coli* network. We compiled a data set of 2,566 known enzymatic and 159 transport reactions across the three kingdoms of life (‘universal reaction set') absent from the *E. coli* model^20^ (see Methods). We next defined a comprehensive sample of the external nutrient space, consisting of 1,776 environments comprised of nutrient sources that can potentially be imported into the network (Supplementary Data 1). We focused on minimal media that differ from each other in a single carbon, nitrogen, sulphur or phosphorus source, thereby maximizing the variability between conditions while remaining computationally feasible (Methods). We determined the phenotypic impact of adding one or more reactions from the universal reaction set to the *E. coli* network in each of these environments using flux balance analysis (FBA)^21^. FBA identifies a steady-state flux distribution that maximizes the production of biomass (a weighted combination of major biosynthetic components) from a given set of available nutrients. This framework successfully predicts the growth capacity of wild-type *E. coli* across nutrient conditions^18^, and it is biologically more realistic than graph-theoretical approaches^22^.

**Figure 1:**
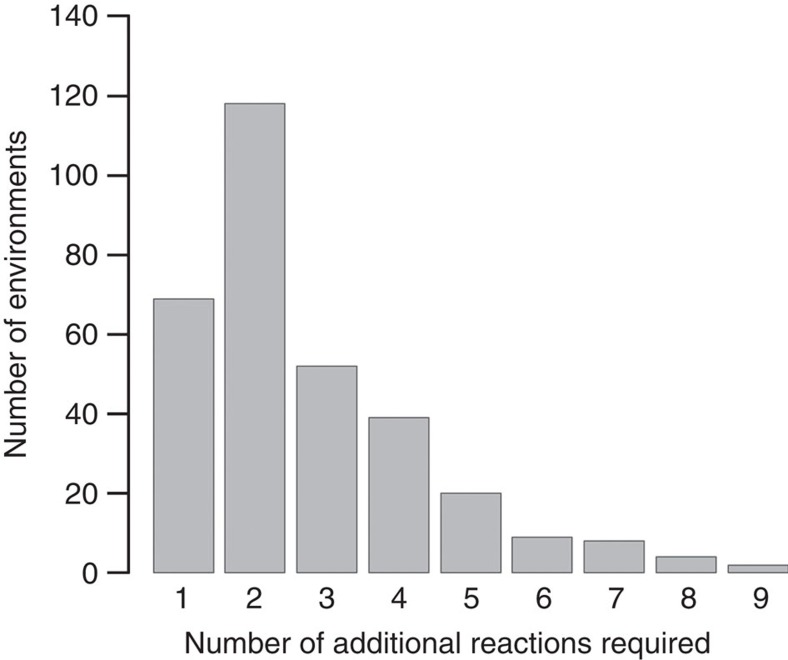
Metabolic innovations in the genotype space around the *E. coli* network. Only few reactions need to be added to the *E. coli* metabolic network to enable growth in metabolic environments where the wild-type cannot grow. The histogram shows the distribution of additional minimal reaction set sizes needed for biomass production in 321 novel nutrient environments.

Before the addition of novel reactions, the reconstructed *E. coli* metabolic network was unable to grow (that is, the rate of biomass production was zero) in 321 environments in which the network expanded by the complete universal reaction set was able to grow (Supplementary Data 1). Using a mixed integer linear programming (MILP) algorithm, we determined the minimal number of reactions from the universal reaction set that need to be added to the *E. coli* network to support growth in these novel environments. Strikingly, growth in additional environments required the addition of only one to three enzymatic and transport reactions in 74% of the cases (239 out of 321 environments; see Fig. 1). In 21.5% of the novel environments, acquisition of only one reaction was sufficient for growth (69 out of 321 environments, see Supplementary Data 2). These results suggest that in the genotype space around the *E. coli* metabolic network, most metabolic innovations are only a few gene acquisition steps away.

### Complex innovations can arise via changing environments

**Figure 2:**
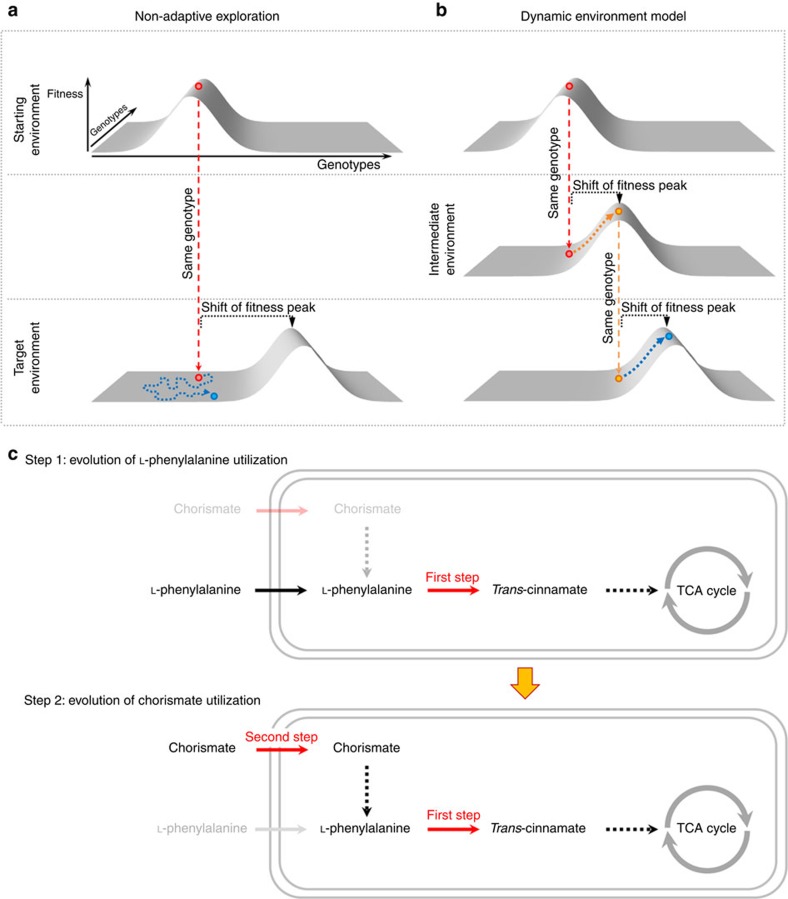
Evolution in varying environments is expected to facilitate the establishment of complex metabolic traits. (**a**, top) Hypothetical fitness landscape over a two-dimensional genotype space. The red genotype is well-adapted, that is, it is located on the fitness peak of this starting fitness landscape. A change to the target environment shifts the fitness peak, so that the red genotype is no longer of high fitness (bottom). Adaptation to the shifted peak now cannot proceed purely through adaptive steps (that is, hill climbing); it requires the non-adaptive exploration of the neutral part of the landscape, illustrated by the yellow dotted line. (**b**) Depicting the same situation, but with an intermediate environment in which the fitness peak is only slightly shifted relative to the starting environment. The red genotype is located at the foot of the shifted fitness peak in this intermediate environment and can thus progress through purely adaptive steps, culminating in the yellow high-fitness genotype. When the environment now changes to the same target environment as in **a**, the blue genotype represents an exaptation, such that it can now progress towards the target fitness peak through purely adaptive steps. While **b** only shows one intermediate environment, the same reasoning applies to more complex scenarios including dynamic landscapes with moving peaks. (**c**) Example from simulated metabolic network expansions. *E. coli* K-12 is unable to utilize chorismate and L-phenylalanine as sole carbon sources. Simulations show that while chorismate utilization demands the simultaneous addition of two reactions to the network, one of these reactions (first step; catalysed by phenylalanine ammonia lyase) also confers L-phenylalanine utilization when added individually.

One can envisage a simple adaptationist hypothesis by which complex metabolic innovations can arise. A metabolic phenotype accessible through the addition of a single reaction may serve as an exaptation^23^ from which metabolic phenotypes that demand the acquisition of multiple reactions can be developed. A major corollary of this hypothesis is that evolutionary adaptation to temporally varying environmental conditions facilitates the expansion of metabolic networks (see also ref. 16). In the parlance of fitness landscapes, varying environments result in dynamic landscapes with moving peaks which can be more easily tracked by hill-climbing evolution (see Fig. 2a,b).

To test the feasibility of the stepwise network expansion scenario, we focused on reaction pairs that are jointly required to provide a fitness benefit in at least one environment (for a list of the 538 such reaction pairs, see Supplementary Data 3). Next, we added each of the corresponding reactions individually into the network and asked whether their presence alone provides a selective advantage across the set of 321 novel environments. Consistent with the hypothesis, we found that in 40% of the 538 growth-promoting reaction pairs, one of the reactions enables growth on its own in at least one environmental setting, which therefore can serve as stepping stones along adaptive trajectories. For example, while the ability to metabolize chorismate demands the simultaneous acquisition of two reactions, one of them also confers L-phenylalanine utilization when added individually to the network (Fig. 2c). We note that many growth-promoting reaction pairs are phenotypically equivalent (that is, confer growth in the same environment) and share the same stepping-stone reaction (Supplementary Data 3). As a result, in total 8.5% of the 118 novel environments that require the simultaneous addition of two reactions becomes accessible through purely adaptive walks.

To more generally assess the potential for exaptation, we examined for each novel environment if its growth-promoting reactions are involved in adaptation to another (intermediate) environment. To this end, for each environment, we enumerated all possible minimal reaction sets that can support growth when added to the *E. coli* network from the universal reaction set. On average, 26% of the alternative minimal reaction sets required for growth in a given environment are also entirely present in at least one minimal growth-promoting reaction set of a second environment. This finding indicates that some of the growth-promoting reaction sets contribute to growth in multiple environments as parts of larger reaction sets. These figures are likely underestimates due to incomplete knowledge of available enzymatic reactions (including promiscuous side activities in the *E. coli* metabolic network^24^) and environmental conditions. We conclude that traversing complex evolutionary trajectories can be facilitated by exaptations when the environment varies.

### Metabolic gene acquisition patterns support the hypothesis

**Figure 3:**
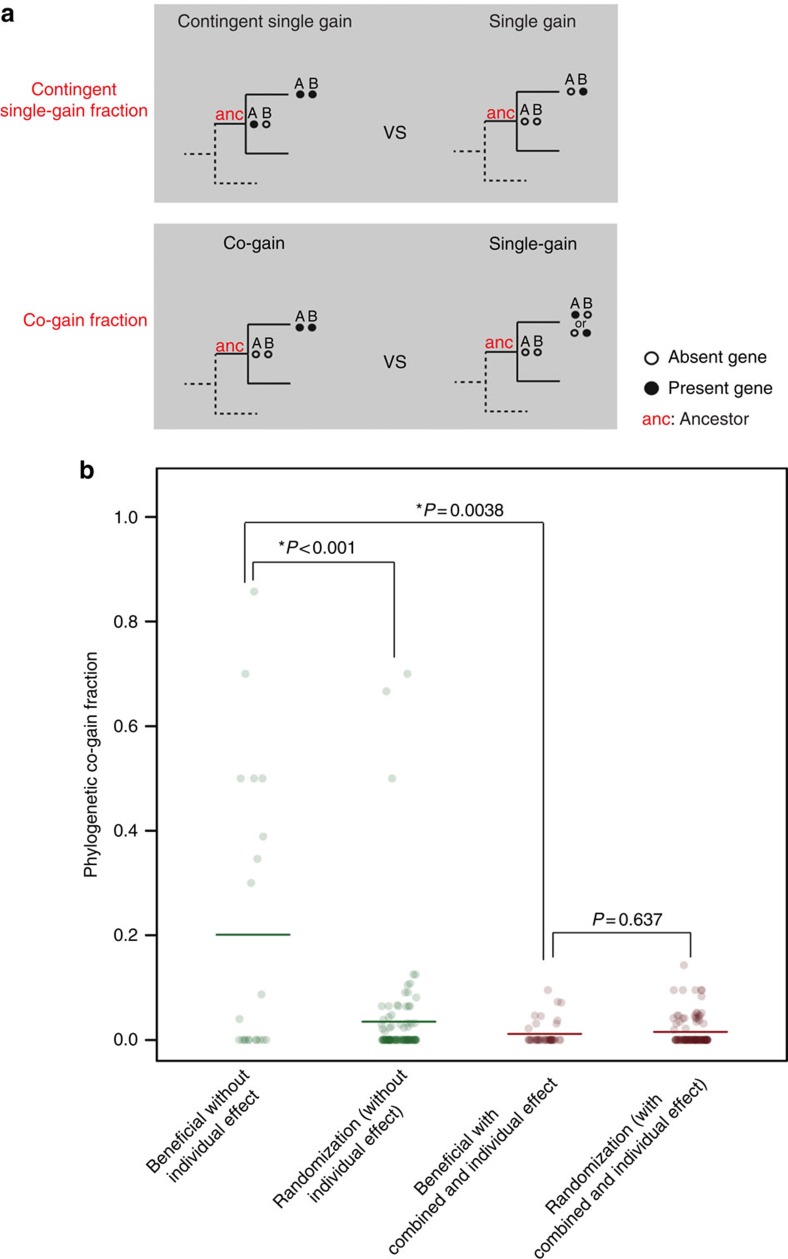
Evolutionary history of gene gains supports the dynamic environment model. (**a**) Schematic representation of the phylogenetic comparisons to study the interdependence between gene-gain events. According to the dynamic environment model, if initial adaptation via a single gain of gene *A* serves as a stepping-stone for complex adaptation via a gain of gene *B*, then acquisition of *B* is expected to occur more frequently with gene *A* being present (contingent gain) compared with *A* being absent in the ancestral branch points of the bacterial tree (upper panel). Furthermore, enzyme pairs that confer a growth benefit only when present together are expected to be more frequently co-gained along branches of the bacterial tree in comparison to a gain of only one of the two (lower panel). Detailed description of the procedures is presented in Methods. (**b**) Phylogenetic co-gain measure (see Methods) of jointly beneficial enzymes based on analysis of hundreds of bacterial genomes. Orthologs of enzyme pairs that are beneficial jointly but not accessible gradually (‘beneficial without individual effect', *N*=21) tend to be co-gained on the same branch of the phylogenetic tree. This trend is statistically significant when compared both with randomized pairs and to enzymes that are growth-promoting as a pair but are accessible gradually through adaptive evolution via varying environments (‘beneficial with combined and individual effect', *N*=40), *P*<0.001 (randomization analysis) and *P*=0.0038 (one-sided Wilcoxon rank test), respectively. In addition, such ‘accessible' pairs are not more likely to be co-gained than expected by chance (*P*=0.637).

The model predicts that acquisition of new metabolic genes during bacterial evolution should be contingent on the presence of other genes providing specific adaptations to intermediate environments. It has been established that a major source of metabolic network expansion is horizontal gene transfer in bacteria^19^,^25^. Genes recently acquired by *E. coli* through horizontal gene transfer confer condition-specific advantages and contribute to growth only in specific environments^19^. To test whether acquisition of an enzyme pair that is potentially accessible via adaptive steps occurs via a defined order, we used genomic data from 943 bacteria to reconstruct gene-gain events along the corresponding phylogeny using parsimony (Fig. 3a, Methods). As expected under the hypothesis, enzymes that are predicted to confer fitness benefits on their own and can hence serve as stepping stones towards two-step adaptations *in silico* tend to be gained on an earlier branch of the phylogenetic tree than their partner enzyme (in 65% of cases, *N*=33, as opposed to 50% expected by chance, *P*=0.037, one-tailed one-sample Wilcoxon signed-rank test, see Methods). We note that this pattern holds for different parameter values of the gene-gain reconstruction procedure (see Supplementary Table 1).

In contrast to such cases, growth-promoting enzyme pairs not accessible gradually are the most likely candidates for co-gain via horizontal gene transfer. In agreement with this expectation, such enzyme pairs show a much higher co-gain fraction, that is, number of co-gain relative to single gain events, compared with random gene pairs and growth-promoting gene pairs predicted to be accessible gradually through adaptive evolution via environmental changes (*P*<0.001, randomization analysis and *P*=0.0038, one-sided Wilcoxon rank test, respectively, *N*=21, Fig. 3b, see Methods). Also consistent with the hypothesis, growth-promoting enzyme pairs that are accessible gradually through adaptive evolution via environmental changes, have very low co-gain fractions that are indistinguishable from that of random gene pairs (*P*=0.64, randomization analysis, *N*=40, Fig. 3b, see Methods). These conclusions are robust to changes in parameter values of the gene-gain reconstruction procedure (see Supplementary Tables 2,3).

### Experimental evolution of a complex metabolic innovation

New metabolic pathways can evolve not only through the acquisition of full-blown enzymes from other organisms but also through the enhancement of weak side activities of existing enzymes^3^,^24^. Thus, a further prediction of the hypothesis is that evolutionary adaptation to a specific nutrient via accumulating mutations in endogenous genes can influence the accessibility of adaptive paths towards the utilization of other nutrients. An early work^26^ suggests that acquiring the ability to grow on ethylene glycol (EG, ethane-1, 2-diol) and propylene glycol (PG, (S)-propane-1, 2-diol), two related carbon sources unavailable for utilization by wild-type *E. coli* K12, might depend on one another in a contingent manner. Specifically, according to the anecdotal report, *E. coli* mutants able to grow on EG could be obtained from mutants that could grow on propylene glycol^26^. Using these phenotypes as a test bed we aimed at directly testing the stepwise metabolic niche expansion scenario by examining (i) whether mutations that enable growth on propylene glycol *per se* increase adaptation rates to EG and (ii) whether the mutations conferring these two distinct growth phenotypes exhibit epistasis on EG, as predicted by the hypothesis.

First, we attempted to isolate mutants that can grow on EG (EG+) or propylene glycol (PG+) from large populations of bacteria (Supplementary Methods). No EG+ or PG+ cells were isolated from ∼10^11^ cells with wild-type mutation rate (Table 1), demonstrating that these substrates demand the acquisition of one or more very rare specific mutations. Next, we employed an *E. coli* strain with an approximately 1,000-fold increased mutation rate^27^. In this case, PG+ cells occurred at a low, but detectable frequency of 1.5 × 10^−9^, but still no EG+ mutants were found (Table 1). As discussed, the evolution of EG utilization might be facilitated by prior adaptation to PG^26^. This was indeed so: EG-utilizing cells were detected in PG+ populations at a frequency of ∼3.8 × 10^−9^ (Table 1), indicating an increase in adaptation rate of at least two orders of magnitude.

**Figure 4:**
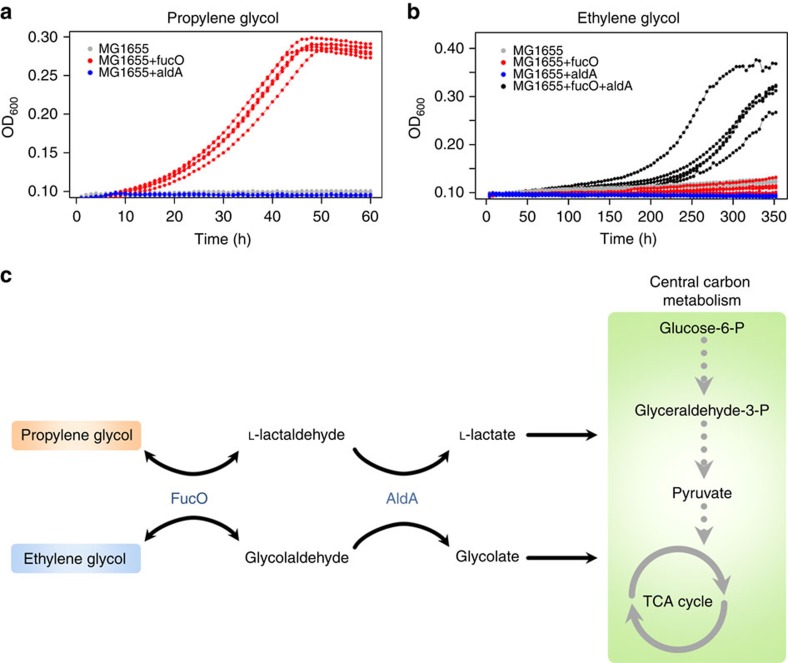
Utilization of propylene glycol increases adaptation rates towards growth on EG in the laboratory. (**a**) Growth curve measurements demonstrating that overexpression of *fucO* (red) is sufficient for growth in propylene glycol. Wild-type MG1655 strain is depicted in grey. OD_600_ measurements of six independent replicates were taken every 60 min. (**b**) Growth curve measurements demonstrating that joint overexpression of both *fucO* and *aldA* is required for growth on EG (black). Neither *fucO* (red) nor *aldA* (blue) can achieve this when overexpressed individually. Wild-type MG1655 strain is depicted in grey. OD_600_ measurements of six independent replicates were taken every 240 min. One replicate population with joint overexpression of *fucO* and *aldA* failed to grow for unknown reason and is not shown. (**c**) Schematic pathway diagram representing the role of FucO and AldA enzymes in the utilization of PG and EG. In the first step, FucO catalyses the oxidation of PG and EG to glycolaldehyde and L-lactaldehyde, respectively. We note that the native activity of FucO operates in the reverse direction by reducing L-lactaldehyde to PG during the catabolism of L-fucose and L-rhamnose. In the next step, AldA oxidizes the products of FucO to hydroxycarboxylic acids which can be wired into central carbon metabolism following further enzymatic modifications. The affinity of AldA for L-lactaldehyde (PG utilization) is higher than for glycolaldehyde (EG utilization)^30^, potentially explaining why growth on EG requires multiple copies of *aldA*.

It has been reported that constitutive activation of *fucO*, a gene encoding an enzyme involved in fucose and rhamnose catabolism, is a prerequisite for growth in PG^28^. We therefore hypothesized that *fucO* upregulation acts as a stepping-stone mutation towards EG utilization. To test this scenario, we overexpressed *fucO* from a strong constitute promoter in wild-type background^29^. As expected, *fucO* overexpression conferred the ability to utilize PG (Fig. 4a). Remarkably, employing a *fucO* overexpressed PG+ strain yielded EG-utilizing cells at a frequency of ∼2 × 10^−8^ (Table 1). As this strain retained a wild-type mutation rate (Supplementary Fig. 1), this finding shows that the ability to metabolize PG *per se* promotes adaptation to EG. Whole-genome sequencing of an EG-utilizing strain suggested that ∼10-fold amplification of a genomic segment encoding *aldA* might underlie EG utilization (Supplementary Table 4). Indeed, simultaneous overexpression of both *fucO* and *aldA* in wild-type background conferred the ability to grow on EG (Fig. 4b) with a growth kinetics akin to the strain adapted to EG (Supplementary Fig. 2). Furthermore, as neither *fucO* nor *aldA* alone conferred growth on EG, this finding provides evidence that the two overexpression mutations act epistatically, as predicted by the stepwise metabolic niche expansion hypothesis.

How do these two enzymes, FucO and AldA, contribute to EG utilization? FucO likely acts on EG in addition to its native substrate to produce glycolaldehyde from EG^26^; AldA, an enzyme with broad substrate specificity, further converts glycolaldehyde to glycolate^30^ (Fig. 4c). Interestingly, in addition to their role in EG metabolism, both enzymes are involved in PG utilization as well, indicating that regulatory rewiring of the same enzyme toolkit can produce multiple qualitatively different phenotypes.

## Discussion

Explaining the origin of evolutionary innovations that require the simultaneous acquisition of multiple mutations, none of which seemingly confer a benefit individually, remains a central challenge in evolutionary biology. On the basis of prior theoretical considerations^11^,^12^,^16^, here we propose that metabolic innovations accessible through the addition of a single reaction serve as stepping stones towards the later establishment of complex metabolic features in another environment. We provided several lines of evidences in support of the hypothesis by focusing on the most well-studied molecular network, cellular metabolism, and by employing three complementary approaches. First, we simulated the adaptation of the *E. coli* metabolic network to novel environments. We revealed that new complex pathways can evolve via the successive acquisition of single biochemical reactions that allow the utilization of specific nutrients. Second, by reconstructing the evolutionary history of gene gains in bacteria, we demonstrated that complex metabolic pathways are indeed often established in a defined order as predicted. Finally, we conducted a laboratory evolution study of *E. coli* adaptation to two novel carbon sources; evolving the ability to utilize one nutrient remarkably facilitated later adaptation to the other. Thus, complex metabolic traits can emerge without the need to invoke neutral exploration of genotype space, a view that is in sharp contrast to non-adaptive scenarios of evolutionary innovation that rely on the accumulation of neutral intermediate mutations^6^,^7^,^31^.

Taken together, our study demonstrates that complex metabolic innovations can evolve by adaptive means through the step-by-step expansion of nutrient utilization capacities. An important prediction is that metabolic innovations should be intertwined in nature: the ability to metabolize certain nutrients should act as a stepping stone towards the utilization of other nutrient sources^32^. A preliminary systems-level analysis based on nutrient utilization of 168 *E. coli* strains^33^ suggests that it may indeed be so (Supplementary Fig. 3). Experimental case studies on the evolution of the catabolism of β-galactoside sugars^34^ and citrate utilization^35^ are also consistent with the scenario, but it remains to be seen how general these findings are. In addition, it is important to note that functionally linked enzymes frequently cluster in the bacterial genome or are encoded in the same operon and tend to be acquired together during evolution^19^. Future systematic works should study the extent to which simultaneous uptake of multiple physiologically linked reactions by horizontal gene transfer speeds up the evolution of metabolic networks.

We speculate that the major barrier to the dynamic environment model of complex adaptation may be the absence of relevant series of environmental conditions. This restriction could be lifted when multiple novel substrates are simultaneously present in a single environment and evolution proceeds by successively acquiring the capacity to utilize them. We emphasize that other conceptually different mechanisms might also contribute to the adaptive expansion of metabolic networks. For example, stepping-stone reactions might evolve as repair processes in an adaptive response to metabolite damage^36^, to degrade toxic environmental chemicals^3^, or to produce novel secondary metabolites^37^.

Our work has important ramifications for understanding genetic interaction networks and the development of industrially useful microbes. First, epistatic interactions between metabolic genes of the same pathway should often be environment-specific: our results suggest that in many cases, one of the genes should provide fitness benefits independently of the other in at least one environment. Large-scale mapping of genetic interactions across a broad range of environmental conditions would provide a direct way to test this prediction^38^. Second, we anticipate that evolutionary engineering of microbes to obtain desired phenotypes could be facilitated by temporally varying the traits under selection^39^.

Finally, our study could have important implications beyond the evolution of metabolism. Earlier studies claimed that varying environments accelerate evolutionary adaptation in genetic circuits and RNA molecules^12^. In computer science, standard genetic algorithms have a tendency to quickly converge to a local solution, and hence frequently fail to identify more promising regions of the search space^40^. Application of dynamically changing ‘environments' offers a natural strategy to maintain the diversity required to explore the adaptive surface^41^.

## Methods

### Reconstruction of the universal reaction set

To study the potential adaptive value of adding new reactions to the *E. coli* metabolic network, we compiled a data set of metabolic reactions reported from species across the three kingdoms of life (universal reaction set) and absent from *E. coli*. First, we mapped the metabolites of the manually curated *E. coli* genome-scale metabolic model^18^ to the Model SEED database^20^ (and http://blog.theseed.org/model_seed/), a comprehensive resource for automatically generated genome-scale metabolic network reconstructions. Because Model SEED does not contain the most recent version (iJO1366 (ref. 42)) of the *E. coli* network reconstruction, we used an earlier version (iAF1260 (ref. 18)) that is widely utilized and has been extensively tested^43^. As a second step, we added all mass-balanced biochemical reactions from the Model SEED database to the *E. coli* model. From this draft network, we removed duplicate reactions. Next, we removed ‘perpetuum mobile' cycles, that is, flux distributions capable of producing energy without consuming any nutrients (see Supplementary Methods and Supplementary Table 5). Finally, we removed unconditionally blocked reactions (that is, those unable to carry a flux under any condition). The resulting curated universal reaction network contains 4,949 metabolic reactions and 444 nutrient uptake reactions, of which 2,566 and 159 are not present in the *E. coli* network, respectively. The universal network is available as a computational Systems Biology Markup Language (SBML) model (Supplementary Data 4).

For more details on the reconstruction of the universal reaction set, see Supplementary Methods.

### Defining novel *in silico* nutrient environments

We first defined a comprehensive set of nutrient environments by starting from a glucose minimal medium for *E. coli*. For each environment, we replaced the carbon (C), nitrogen (N), phosphate (P) or sulfur (S) source by an alternative one. To obtain a list of environments that is both representative of novel nutrient compounds and computationally tractable, we focused on only those growth media that differed from glucose minimal medium by one compound instead of enumerating all possible combinations of C, N, P and S-sources, as in previous works^24^,^31^. Although this approach does not take into account more complex conditions, it allowed us to focus on single C, N, P and S-sources and to maximize the variability between conditions. See Supplementary Data 1 for the list of resulting 1,776 conditions.

Next, we determined the viability of both the *E. coli* network and the universal network across these conditions using FBA^21^. A network was deemed inviable in a given environment if its maximum biomass production was zero. Before adding novel reactions, the reconstructed *E. coli* metabolic network was unable to grow in 321 environments in which the network expanded by the universal reaction set allowed growth (Supplementary Data 1). We considered these 321 conditions as the set of available novel environments to which *E. coli* can possibly adapt by adding reactions from other species.

### Finding growth-promoting reaction sets in new environments

To calculate the minimum number of active, non-coli reactions in a particular environment we applied a MILP-based algorithm on the universal metabolic model similar to the problem of finding the shortest elementary flux mode^44^. The basis of the MILP problem was the steady-state assumption:


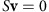


Where *S* is the stoichiometric matrix and **v** is the flux vector for all reactions. The reactions of the model were handled differently depending on whether they are part of the *E. coli* model or they can be added to the coli model during evolution. The flux constraints on the *E. coli* reactions were the same as in FBA:


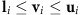


Next, for each environment in which the universal network was viable but the wild-type *E. coli* network was not able to grow we set the nutrient uptake constraints to mimic the environment (**l**_*i*_ of the exchange reactions). The lower bound of the biomass production reaction was then constrained to 10^−4^ as the minimal growth requisite:


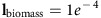


The reversible non-coli reactions of the universal network were decomposed into two opposing irreversible reactions. This way the fluxes of the non-coli reactions can only take positive values. Let *N′* be the number of non-coli reactions. We assigned a binary variable to each non-coli reaction, **b**_i_, which tells whether the non-coli reaction r′_*i*_ *(i=1, …, N′)* is active (**b**_*i*_*=1)* or not (**b**_*i*_*=0)*. The following equations ensure these rules:


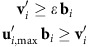


Where **v**′_*i*_ is the flux and **u**′_*i*_ is the maximal possible flux of reaction r′_*i*_, while *ɛ* is the minimal flux value (in our calculations *ɛ=*10^−8^). Also to avoid having two opposing reactions derived from the same reversible reaction being active simultaneously we introduced the following constraint:


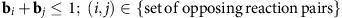


Finally, the objective of the MILP problem was to minimize the active non-coli reactions:


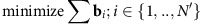


The result of this minimization is the minimum number of non-coli reactions need to be added to the coli model to allow growth in a particular environment.

### Enumerating all possible minimal reaction sets *in silico*

The MILP optimization model described above not only provide the minimal number of reactions that support growth in new environments but also the list of the non-coli reactions involved in this solution: one of the minimal reaction sets. However, multiple equivalent minimal sets might exist for any given environment. To identify another minimal reaction set we extended the MILP problem with a new constraint which prevents the algorithm to find the same solution again:


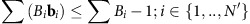


Where *B*_*i*_ is the binary solution of the first minimal reaction set, and *B*_*i*_ equals to 1 or 0 if reaction r′_*i*_ was active or inactive in the first solution, respectively. This constraint is fulfilled only if the two solutions differ in at least one active reaction. We can harvest more minimal reaction sets in an iterative way where after each solution we add a new constraint and we run the algorithm again. Our algorithm stopped when the new solution had more active reactions than the size of the minimal reaction sets, that is, when we collected all minimal reaction sets. This algorithm is based on the method of finding the k-shortest elementary flux modes^44^.

### Defining growth-promoting reaction pairs using modelling

To systematically test the dynamic environment model, we investigated all possible two-step adaptation scenarios. First, we inactivated all non-coli reactions in the universal reaction network. Next, we activated two non-coli reactions at a time and applied FBA to calculate the fitness of the model in each environment where the native *E. coli* model cannot grow. By repeating this procedure we probed all possible reaction pairs in the universal reaction set and identified those that provide growth in at least one environmental condition (3,290,895 reaction pairs in total, 538 are beneficial in at least one condition). As a next step, we determined if the identified two-reaction adaptations can be accessed by the consecutive addition of single beneficial reactions to the network, that is, whether at least one of the two reactions provide a fitness benefit on its own in any of the environments. For this purpose, we repeated the above procedure but instead of activating reaction pairs we activated single reactions and evaluated their fitness effect across environments using FBA. The list of 538 reaction pairs and corresponding environments can be found in Supplementary Data 3.

### Software and computation used in metabolic network analyses

All simulations were implemented in GNU R (ref. 45) using the sybil package for constraint-based modelling^46^. As optimizer for linear programming and MILP we used ILOG CPLEX 12.5. The linear programming was done on a 64-bit Ubuntu Linux system with an Intel Core-i7 quadcore processor. MILP problems were solved on a Red Hat Enterprise Linux Server release 6.2 with 96 Intel Xeon central processing units.

### Phylogenetic analysis of gene-gain events

To investigate contingent gain and co-gain in the evolutionary history of genes, we first generated the phylogenetic presence and absence profile across the present-day species for each reaction by mapping the profiles from gene to reaction level. Presence and absence profiles of orthologous genes across 943 bacterial species were obtained from EggNOG v3.0 (ref. 47). Reactions catalysed by enzyme complexes consisting of multiple gene products (‘AND' relationships) are considered to be present in a species only when all genes of the complex are present in the genome. Reactions catalysed by isoenzymes (‘OR' relationships) are considered to be present when at least one isoenzyme is encoded in the genome.

Next, we inferred the most parsimonious ancestral presence/absence states of each reaction by using a phylogenetic tree of the 943 eubacteria, retrieved from STRING v9.05 (http://string905.embl.de/newstring_download/species.tree.v9.05.txt) (ref. 48). Reaction presence and absence states across branch points along the phylogenetic tree, that is, the ancestral states, are calculated by using the tree and the present-day presence/absence state of the reaction. The ancestral state is inferred by minimizing the number of gene-gain and loss events across the tree that matches the present-day state. Such a maximum parsimony strategy is commonly used as it allows for the analysis of gene histories on a genome-wide scale in a computationally efficient manner, and has shown to be successful in explaining patterns in genome content and evolution^19^,^49^,^50^. Calculations were carried out using PAUP^51^ with a gain/loss penalty ratio of 2/1 (ref. 52) and a delayed transition assumption (DELTRAN)^49^. We note that our results are robust against variations in PAUP parameter values (see Supplementary Tables 1–3).

### Contingent gain analysis

For each stepping-stone reaction pair A–B, A is defined as the reaction that is beneficial in a given nutrient environment without B, while a gain of B is only beneficial in another environment when A is already present. For each A–B pair we calculated the phylogenetic contingent gain fraction (*f*), defined as *f=p*1/(*p*1+*p*2), where *p*1 is calculated by dividing the number of evolutionary events where B is gained in the descendent (d) when A is already present in the ancestor (a) (a10_d11) by the total number of all possible gain and loss scenarios taking place in the descendant when A is present but B is absent in the ancestor (a10_dXX, where X*=*0 or 1), and p2 is calculated by dividing the number of evolutionary events where B is gained in the descendant when A is absent in the ancestor (a00_d01) by the total number of all possible gain and loss scenarios taking place in the descendant when both A and B are absent in the ancestor (a00_dXX, where X*=*0 or 1). The observed distribution of fractions was then compared with the null-hypothesis that a gain of B is independent of the presence of A, that is, *f*=0.5, using a one-tailed one-sample Wilcoxon signed-rank test.

### Co-gain analysis

For the phylogenetic co-gain analysis we calculated for reaction pairs the co-gain fraction, defined as *f=n*1/(*n*1+*n*2), where *n*1 is the number of evolutionary events where both reactions were absent in the ancestor (a) and both were gained in the descendent (d) (a00_d11), and *n*2 is the number of evolutionary events where both reactions were absent in the ancestor and only one was gained in the descendent (a00_d10 or a00_d01). We compared the fractions (*f*) from reaction pairs that are predicted to be beneficial for growth only when they are simultaneously gained, referred to as ‘beneficial without individual effect', with the fractions from reaction pairs that are beneficial for growth in a specific environment when co-gained, but at least one of the reactions is also beneficial on its own in a different environment (beneficial with combined and individual effect) (see Fig. 3b in main text). A one-sided Wilcoxon rank test was used. In addition, we compared the fractions from ‘beneficial without individual effect' reaction pairs with the expected co-gain fraction by chance (randomization (without individual effect)). To do that, we broke the pairing between reactions and shuffled the reactions into new pairs, thereby generating a new list of gene pairs. This was repeated 1,000 times. Then we determined for each of the 1,000 reaction pair list if the mean co-gain fraction is higher than that of the ‘beneficial without individual effect' and summed these (n1). *P*-value was calculated as *P*=(*n*1+1)/1,001. The randomization analysis was also carried out for reaction pairs that are beneficial for growth in a specific environment when co-gained, but at least one of the reactions is also beneficial on its own in a different environment (beneficial with combined and individual effect versus randomization (combined and individual effect)).

### Strains and plasmids and primers for laboratory adaptation

*E. coli* K-12 MG1655 was considered as the wild-type strain in our experiments. MG1655 mutD5 was constructed using a suicide plasmid-based genome engineering method incorporating a C->T mutation at position 236,110 on the genome (within the *dnaQ* gene) resulting in a T15I mutation of the encoded enzyme described previously^27^. Standard steps and plasmids (pST76-A, pSTKST) of this methodology have been described^53^. Briefly, an approximately 800-bp-long targeting DNA fragment carrying the desired point mutation in the middle was synthesized by PCR, then cloned into a thermosensitive suicide plasmid (pST76-A). This plasmid construct was then transformed into the cell, where it was able to integrate into the chromosome by way of a single crossover between the mutant allele and the corresponding chromosomal region. The desired co-integrates were selected by the antibiotic resistance carried on the plasmid at a non-permissive temperature for plasmid replication (42 °C). Next, the pSTKST helper plasmid was transformed, then induced within the cells, resulting in the expression of the I-SceI meganuclease enzyme, which cleaves the chromosome at the 18 bp recognition site present on the integrated plasmid. The resulting chromosomal gap is repaired by way of RecA-mediated intramolecular recombination between the homologous segments in the vicinity of the broken ends. The recombinational repair results in either a reversion to the wild-type chromosome, or in a markerless allele replacement, which can be distinguished by sequencing the given chromosomal region. See Supplementary Table 6 for the primers used for the mutation construction.

For the overexpression of FucO, the pCA24N plasmid containing the *fucO* gene was selected from the ASKA library^29^ and isolated from the host strain, then electroporated into the MG1655 strain. Overexpression of the gene was achieved by the addition of 50 μM IPTG.

For the simultaneous overexpression of *fucO* and *aldA*, the chloramphenicol resistance cassette (Cm^R^) of the pCA24N-*aldA* plasmid from the ASKA library was exchanged to the kanamycin resistance marker (Km^R^), resulting in pCA24N-*aldA*-Km. The pCA24N-*aldA* plasmid was first linearized by inverse PCR amplification using the pCA24N_frame_1 and pCA24N_frame_2 primer pair flanking the Cm^R^ cassette. The PCR product was treated with DpnI for 60 min at 37 °C and purified using the DNA Clean & Concentrator-5 Kit (Zymo Research #D4004). The Km^R^ marker was PCR amplified from a pSTKST template using the ASKA-Gibson_Kan_Fw and ASKA-Gibson_Kan_Rev primers. The PCR fragment was then isolated from 1% agarose gel using the GeneJET Gel Extraction Kit (Thermo Scientific #K0691). The resulting DNA fragments were assembled using Gibson assembly cloning (Gibson Assembly Master Mix, New England Biolabs #E2611), according to the manufacturer's protocol, then electroporated into electrocompetent *E. coli* DH10B cells. Correct assemblies were verified by colony PCR using the ASKA-S2 and aldA-1 primer pair. Sequences of primers used in this construction are listed in Supplementary Table 7.

### Media used in laboratory adaptation

Minimal salts (MS) medium was used as described previously^34^, supplemented either with 0.4% glycerol, 30 mM (S)-propane-1, 2-diol (propylene glycol, PG), or 30 mM ethane-1, 2-diol (EG). Antibiotics were employed in the following working concentrations: 50 μg ml^−1^ ampicillin (Ap), 25 μg ml^−1^ chloramphenicol (Cm) and 25 μg ml^−1^ kanamycin (Km).

### Adaptation of strains for growth on PG and EG

Three replicates of each individual strain were started from single colonies grown on MS+0.4% glycerol agar plates (with Cm added where the *fucO* overexpression plasmid was present) at 30 °C. An MG1655 strain carrying the pCA24N-*fucO* plasmid was previously found to grow at 30 °C in 2 ml MS media supplemented with 30 mM PG (with 25 μg ml^−1^ Cm and 50 μM IPTG added). This culture was subsequently plated onto MS+0.4% glycerol (+Cm) agar plates, from which the PG+colonies, starters for selection for EG-utilization, were isolated. We opted for glycerol as a base carbon source to avoid catabolite repression (that is, the inhibition of utilization of various other carbon sources) as in ref. 28. Starter cultures were then grown in 2 ml MS+0.4% glycerol (+Cm where needed), from which 250 μl was then transferred to 25 ml fresh liquid MS media+0.4% glycerol (and Cm where needed). Cultures were grown to stationary phase at 30 °C, after which total cell count was determined by plating of appropriate dilutions onto MS+0.4% glycerol agar plates. The remainder of the cultures were then harvested and resuspended in 400 μl MS media without carbon source and finally plated in two halves onto MS agar plates supplemented with either 30 mM PG or 30 mM EG (with Cm and 50 μM IPTG added where the *fucO* overexpression plasmid was present). Plates were then incubated at 30 °C for 40 days after which adapted colonies were counted and isolated. The plates were placed in plastic bags for the duration of the incubation to prevent significant drying of the agar media. Rates of adaptive mutations were calculated based on three replicate experiments as follows. When adapted colonies were observed, we simply calculated the average ratio of the number of adapted colonies per total cell number. In cases where no growing colonies were obtained, we calculated an upper limit to the adaptive mutation rate following the approach presented in ref. 35. Specifically, we made use of the fact that the Poisson distribution has a 5% probability of yielding zero events when the expected number of events is three. Thus, assuming no more than three adaptive mutations among all the cells tested in the three replicate experiments gives an upper bound on the adaptive mutation rate per cell per generation.

### Growth curve measurements

Individual colonies of strains MG1655, MG1655+pCA24N-*fucO*, MG1655+pCA24N-*aldA*-Km and MG1655+pCA24N-*fucO*+pCA24N-*aldA*-Km were grown and isolated from MS+0.4% glycerol plates carrying the desired antibiotic for the given plasmids. Starter cultures from single colonies were grown in 5 ml liquid MS media supplemented with 0.4% glycerol, as well as 50 μM IPTG and 25 μg ml^−1^ Cm and/or 25 μg ml^−1^ Km in the case of plasmid-harbouring strains. Cultures were grown until saturation after which 10 ml MS media supplemented with 30 mM of either PG or EG as well as 50 μM IPTG and 25 μg ml^−1^ Cm and/or 25 μg ml^−1^ Km where needed, were inoculated with the overnight cultures at a 100 × dilution. A total of 100 μl of these samples were then placed in six separate wells on a 96-well tissue culture plate (Jet Biofil), and placed in a PowerWave XS2 (BioTek) microplate spectrophotometer and grown at 30 °C. The edges of the plate were sealed with Breathe-Easy gas permeable sealing membrane (Diversified Biotech) to prevent evaporation.

### Mutation rate measurements

We estimated mutation frequencies of BW25113 (wild-type) and BW25113 overexpressing the FucO protein from the pCA24N_fucO plasmid. Briefly, cells resistant to rifampicin (carrying mutations in *rpoB* (ref. 54)) were selected and counted. After overnight growth at 37 °C, ten tubes of 1 ml LB (+25 μg ml^−1^ chloramphenicol in the case of pCA24N_fucO carrying samples) were inoculated with approximately 10^4^ cells each. FucO overexpression was induced by adding 50 μM IPTG after 2 h of growth, and cultures were grown to early stationary phase, all at 37 °C. Appropriate dilutions were spread onto non-selective LB agar plates and LB agar plates containing rifampicin (100 μg ml^−1^). The samples were incubated at 37 °C and colony counts were performed after 24 or 48 h, respectively. Mutation rates were calculated with the Ma–Sandri–Sarkar maximum-likelihood method^55^ using the FALCOR web tool^56^.

### Ion Torrent library construction for whole-genome sequencing

Fragment libraries were constructed from purified genomic DNA using NEBNext Fast DNA Fragmentation & Library Prep Set for Ion Torrent (New England Biolabs) according to manufacturer's instructions. Briefly, genomic DNA was enzymatically digested and the fragments were end-repaired. Ion Xpress Barcode Adaptors (Life Technologies) were than ligated and the template fragments size-selected using AmPure beads (Agencourt). Adaptor ligated fragments were then PCR amplified, cleaned-up using AmPure beads, quality checked on D1000 ScreenTape and Reagents using TapeStation instrument (Agilent) and finally quantitated using Ion Library TaqMan Quantitation Kit (Life Technologies). The library templates were prepared for sequencing using the Life Technologies Ion OneTouch protocols and reagents. Briefly, library fragments were clonally amplified onto Ion Sphere Particles (ISPs) through emulsion PCR and then enriched for template-positive ISPs. More specifically, PGM emulsion PCR reactions utilized the Ion OneTouch 200 Template Kit (Life Technologies), and as specified in the accompanying protocol, emulsions and amplification were generated using the Ion OneTouch System (Life Technologies). Enrichment was completed by selectively binding the ISPs containing amplified library fragments to streptavidin-coated magnetic beads, removing empty ISPs through washing steps, and denaturing the library strands to allow for collection of the template-positive ISPs. For all reactions, these steps were accomplished using the Life Technologies ES module of the Ion OneTouch System. Template-positive ISPs were deposited onto the Ion 318 chips (Life Technologies); finally, sequencing was performed with the Ion PGM Sequencing Kit (Life Technologies).

### Ion PGM sequencing data processing and mutation calling

The PGM sequencing data was processed using Ion Torrent Suite v4.2.1 in order to perform signal processing and base calling. Read mapper module of Torrent Suite (tmap) was used to align raw reads to the *E. coli* K12 MG1655 genome sequence (U00096.3). Torrent Variant caller (tvc) module of Torrent Suite was subsequently applied to detect single nucleotide mutations as well as small in/del variants. Variant caller was programmed to run in high stringency mode requesting at least 12 × read coverage and at least 66% mutation frequency. Only those variants were taken into account that were supported by sequencing on both strands. BAM alignment files were imported in CLC Genomics Workbench v7.5.1 (CLCBio) and variant regions were manually inspected in all strains. Large genomic rearrangements (deletions or amplifications with lengths above 10 kb) were manually identified using CLC Genomics Workbench Tool.

Sequencing data of the ancestral and evolved strains are deposited in the NCBI SRA database (accession numbers SRX1167076 and SRX1167031).

## Additional information

**Accession codes:** Sequencing data of the ancestral and evolved strains are deposited in the NCBI SRA database with accession codes SRX1167076 and SRX1167031.

**How to cite this article:** Szappanos, B. *et al*. Adaptive evolution of complex innovations through stepwise metabolic niche expansion. *Nat. Commun.* 7:11607 doi: 10.1038/ncomms11607 (2016).

## Supplementary Material

Supplementary Information Supplementary figures 1-3, supplementary tables 1-7, supplementary methods, supplementary references

Supplementary Dataset 1 lists the set of 1776 environmental conditions defined in the present study and the in silico biomass flux for both the E. coli and the universal reaction networks.

Supplementary Dataset 2 lists the reactions which enable in silico growth in a condition where the E. coli metabolic network cannot grow

Supplementary Dataset 3 lists all reaction pairs which can confer a novel nutrient utilization phenotype when added to the E. coli metabolic network

Supplementary Dataset 4 the universal metabolic network in SBML format

## Figures and Tables

**Table 1:** Adaptation frequencies of different strains to PG and EG.

**Strain**	**Frequency of cells growing on PG**	**Frequency of cells growing on EG**
MG1655	Up to 1.6 × 10^−11^	Up to 1.6 × 10^−11^
MG1655 mutD5	1.5 × 10^−9^	Up to 3.1 × 10^−11^
MG1655 mutD5 adapted to PG	Grows on PG	3.8 × 10^−9^
MG1655+*fucO* overexpressed	Grows on PG	2.1 × 10^−8^

EG, ethylene glycol; PG, propylene glycol.

MG1655 is the reference wild-type strain, while MG1655 mutD5 refers to a strain with an approximately 1,000-fold increased mutation rate. Values are averages of three parallel replicates when PG+ or EG+ cells were observed and upper estimates^35^ when no growing cells were obtained (see Supplementary Methods).
