# Supplementary material for: Adaptive evolution of complex innovations through stepwise metabolic niche expansion
Source: Nat Commun. 2016 May 20;7:11607. doi: 10.1038/ncomms11607 (PMC5411730; doi:10.1038/ncomms11607)
Supplement: Supplementary Information — Supplementary figures 1-3, supplementary tables 1-7, supplementary methods, supplementary references [file ncomms11607-s1.pdf]

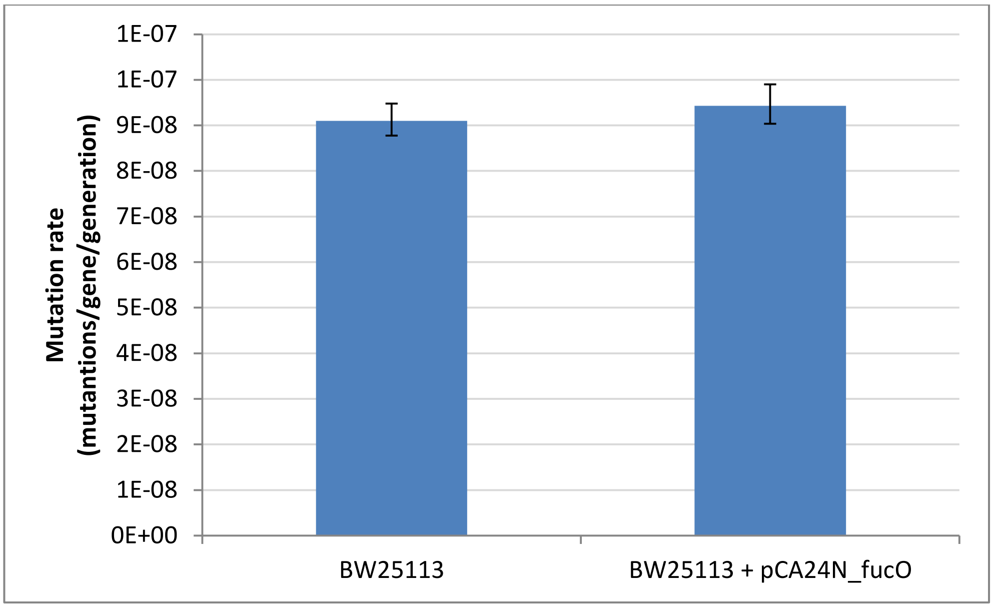

Supplementary Figure 1: Mutation rates of BW25113 (wild-type) and BW25113 overexpressing the FucO protein from the pCA24N_fucO plasmid.

Mutation frequencies were estimated by selecting and counting cells resistant to rifampicin. Mutation rates were calculated with the Ma-Sandri-Sarkar maximum-likelihood method [1] using the FALCOR web tool [2]. Error bars represent 95 % confidence intervals of 10 parallel samples each. See Methods for details.

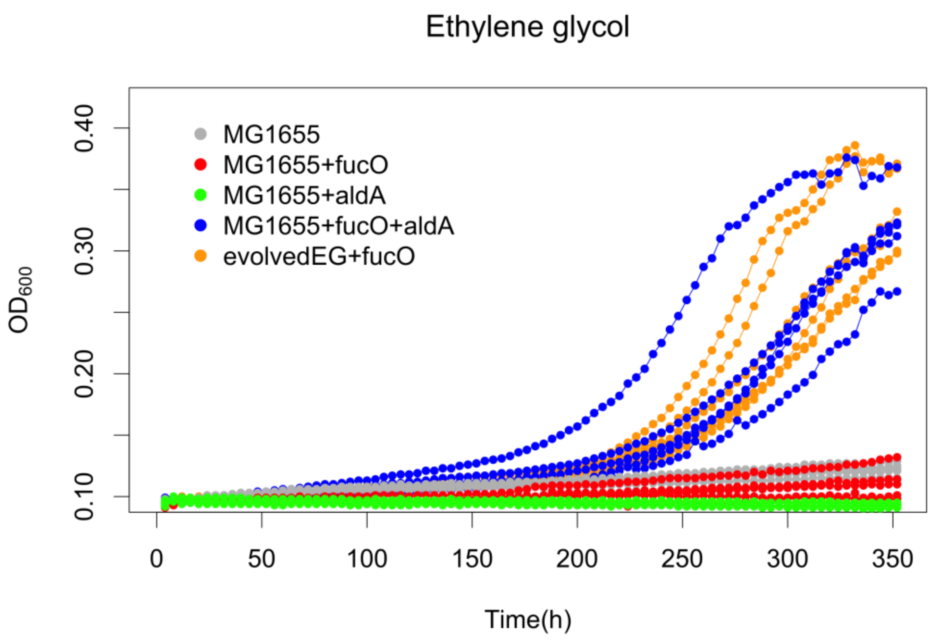

Supplementary Figure 2: Joint overexpression of *fucO* and *aldA* is sufficient to confer growth on ethylene glycol.

Growth curve measurements demonstrating that joint overexpression of both *fucO* and *aldA* is required for growth on ethylene glycol (blue). This double overexpression strain grows as well as a strain directly selected to grow on ethylene glycol from a *fucO* overexpression background (orange). Neither *fucO* (red) nor *aldA* (green) can achieve this when overexpressed individually. Wild-type MG1655 strain is depicted in grey. OD_{600} measurements of six independent replicates were taken every 240 minutes. One replicate population with joint overexpression of *fucO* and *aldA* failed to grow for unknown reason and is not shown.

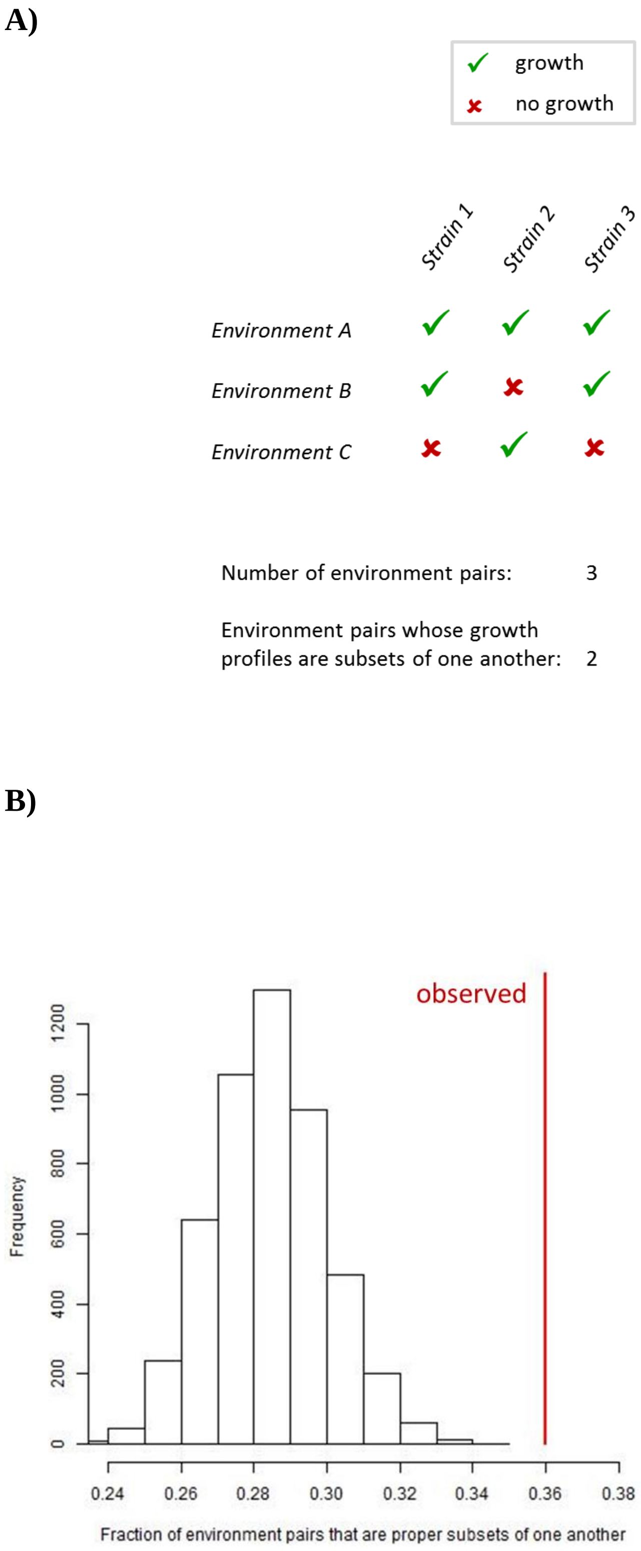
A)
growth
no growth
Strain 1
Strain 2
Strain 3
Environment A
Environment B
Environment C
Number of environment pairs: 3
Environment pairs whose growth profiles are subsets of one another: 2
B)
observed
Frequency
0
200
400
600
800
1000
1200
0.24
0.26
0.28
0.30
0.32
0.34
0.36
0.38
Fraction of environment pairs that are proper subsets of one another

Supplementary Figure 3: Intertwined nutrient utilization capabilities across *E. coli* strains.

A) If the capacity to evolve to environment B relies on the prior capacity to grow in environment A, then the metabolic phenotype to grow in A should be more widespread, and species with capacity to grow in B but not in A should be relatively rare. Thus, the set of species growing in B is expected to be a subset of species growing in A. The figure schematically represents how the degree of non-independence of growth phenotypes is calculated here. In this toy example, strains that can grow in environment B also have the capacity to grow in A, hence the growth profile of strains in B is a proper (i.e., strict) subset of that in A. Similarly, the capacity to grow in C is a proper subset of growth in A. In contrast, the set of strains that grow in C is not a subset of strains that grow in B (and vice versa), hence B and C are not subsets of each other. Thus, 2 out of 3 environment pairs are proper subsets of one another. **B)** To test whether nutrient utilization phenotypes are intertwined as predicted by the dynamic environment model, we analyzed a previously compiled dataset on utilization of 95 carbon sources by 168 closely related *E. coli* and *Shigella* strains [3]. To estimate their metabolic phenotypic diversity, the authors assessed their growth performance on each carbon source. Consistent with the dynamic environment model, the distribution of nutrient utilization phenotypes across the 168 strains was far from being random: we found a significant excess of nutrient pairs where the set of strains utilizing one of the two nutrients was a subset of those utilizing the other one ($P<10^{-3}$ as assessed by randomization). The plot shows the fraction of environment pairs for which the growth profile of one environment is a proper subset of the growth profile of the other, as observed in real (red vertical line) and in 5,000 randomized data (histogram).

Supplementary Tables

Supplementary Table 1. Sensitivity analysis of the gene gain order test.

Gain/Loss penalty	Contingent single gain fraction with DELTRAN (P-value)	Contingent single gain fraction with ACCTRAN (P-value)
5/1	0.799 (0.006)	0.705 (0.018)
4/1	0.703 (0.018)	0.706 (0.018)
3/1	0.662 (0.035)	0.721 (0.0065)
2/1	0.651 (0.037)	0.620 (0.044)

The table shows the computed mean contingent single gain fractions for different PAUP parameters: gain/loss penalty, delayed transformation (“DELTRAN”), i.e., present/absent status changes are assigned along branches closest to the end leaves of the phylogenetic tree and accelerated transformation (“ACCTRAN”), i.e., present/absent status changes are assigned along branches closest to the root of the phylogenetic tree. P-values are listed between brackets and are calculated from comparing the observed contingent single gain fraction with that expected by chance (i.e., no contingency, f=0.5, see also Methods in main text).

Supplementary Table 2. Sensitivity analysis of the gene co-gain test / DELTRAN.

	**DELTRAN**			
Gain/Loss penalty	Beneficial without individual effect	Randomization for pairs without individual effect (P-value; comparison to Beneficial without individual effect)	Beneficial with combined & individual effect (P-value; comparison to Beneficial without individual effect)	Randomization for pairs with combined & individual effect (P-value; comparison to Beneficial with combined & individual effect)
5/1	0.153	0.0115 (< 0.001)	0 (0.0001)	0.0007 (1)
4/1	0.152	0.0137 (<0.001)	0.0031 (8.58e-05)	0.001 (0.432)
3/1	0.182	0.0138 (< 0.001)	0.0088 (0.0007)	0.01 (0.549)
2/1	0.201	0.028 (< 0.001)	0.0113 (0.0038)	0.014 (0.637)

The table shows the computed mean co-gain fraction for different PAUP parameters: gain/loss penalty and delayed transformation ("DELTRAN"), i.e., present/absent status changes are assigned along branches closest to the end leaves of the phylogenetic tree. P-values are listed between brackets. These p-values are calculated from a comparison to another group, as indicated between the brackets.

Supplementary Table 3. Sensitivity analysis of the gene co-gain test / ACCTRAN.

	**ACCTRAN**			
Gain/Loss penalty	Beneficial without individual effect	Randomization for pairs without individual effect (P-value; comparison to Beneficial without individual effect)	Beneficial with combined & individual effect (P-value; comparison to Beneficial without individual effect)	Randomization for pairs with combined & individual effect (P-value; comparison to Beneficial with combined & individual effect)
5/1	0.116	0.012 (< 0.001)	0 (0.00023)	0.0006 (1)
4/1	0.198	0.016 (< 0.001)	0.0028 (4.95e-05)	0.0015 (0.212)
3/1	0.201	0.024 (< 0.001)	0.0054 (1.98e-05)	0.009 (0.720)
2/1	0.174	0.038 (< 0.001)	0.053 (0.048)	0.040 (0.173)

The table shows the computed mean co-gain fraction for different PAUP parameters: gain/loss penalty and accelerated transformation (“ACCTRAN”), i.e., present/absent status changes are assigned along branches closest to the root of the phylogenetic tree. P-values are listed between brackets. These p-values are calculated from a comparison to another group, as indicated between the brackets.

Supplementary Table 4. Whole-genome sequencing results of an EG-adapted line carrying the *fucO*-overexpression plasmid..

Mutation	Genome position	Possible significance
lacY Asp68Glu missense variant (C → G)	362199	Fine-tuning of *fucO* overexpression from IPTG-inducible promoter [4]
rpoC inframe deletion (GAACGTGTA)	4186977	Unknown
15.6 Kbp deletion	1962900 – 1978500	Elimination of flagellar apparatus unnecessary in the applied growth conditions
2x amplification of 76.774 Kbp region	1886126 – 1962900	Unknown
10x amplification of 68.663 Kbp region	1482623 – 1551286	Amplification of *aldA* gene which is required for EG utilization

Mutations represent differences between an evolved line adapted to grow on EG and the ancestral K-12 MG1655-derived strain carrying the pCA24N-*fucO* plasmid. Coordinates are given for the genomic sequence of *E. coli* K-12 MG1655[5]

Supplementary Table 5: Energy dissipating reactions that are employed to detect 'perpetuum mobile' cycles in the universal reaction network

Reaction equation with metabolite ids	Reaction equation with metabolite names
cpd00002[c] + cpd00001[c] -> cpd00008[c] + cpd00067[c] + cpd00009[c]	ATP + H2O -> ADP + H+ + Phosphate
cpd00052[c] + cpd00001[c] -> cpd00096[c] + cpd00067[c] + cpd00009[c]	CTP + H2O -> CDP + H+ + Phosphate
cpd00038[c] + cpd00001[c] -> cpd00031[c] + cpd00067[c] + cpd00009[c]	GTP + H2O -> GDP + H+ + Phosphate
cpd00062[c] + cpd00001[c] -> cpd00014[c] + cpd00067[c] + cpd00009[c]	UTP + H2O -> UDP + H+ + Phosphate
cpd00068[c] + cpd00001[c] -> cpd00090[c] + cpd00067[c] + cpd00009[c]	ITP + H2O -> IDP + H+ + Phosphate
cpd00004[c] -> cpd00003[c] + cpd00067[c]	NADH -> NAD + H+
cpd00005[c] -> cpd00006[c] + cpd00067[c]	NADPH -> NADP + H+
cpd00982[c] -> cpd00015[c] + 2 cpd00067[c]	FADH2 -> FAD + 2 H+
cpd01270[c] -> cpd00050[c] + 2 cpd00067[c]	FMNH2 -> FMN + 2 H+
cpd15561[c] -> cpd15560[c] + 2 cpd00067[c]	Ubiquinol-8 -> Ubiquinone-8 + 2 H+
cpd15499[c] -> cpd15500[c] + 2 cpd00067[c]	Menaquinol 8 -> Menaquinone 8 + 2 H+
cpd15353[c] -> cpd15352[c] + 2 cpd00067[c]	2-Demethylmenaquinol 8 -> 2-Demethylmenaquinone 8 + 2 H+
cpd00001[c] + cpd00022[c] -> cpd00067[c] + cpd00029[c] + cpd00010[c]	H2O + Acetyl-CoA -> H+ + Acetate + Coenzyme A
cpd00023[c] + cpd00001[c] -> cpd00024[c] + cpd00013[c] + 2 cpd00067[c]	L-Glutamate + H2O -> 2-Oxoglutarate + NH4 + 2 H+
cpd00067[p] -> cpd00067[c]	H+(periplasm) -> H+(cytoplasm)

Supplementary Table 6: Primers used for the mutation construction

Primer name	Sequence (5'-3')
dnaQ -MA	ggaattcatacggttgttggtggtgc
dnaQ-MBF	tctcgataccgaaatcaccggtatgaacc
dnaQ-MBR	ggttcataccggtgatttcggtatcgaga
dnaQ-MC	cggatcctagcattgataacggactc
dnaQ-MD	gcgacaatagcggccatca
dnaQ-ME	cctgcaagcagcgagaata

Supplementary Table 7: Primers used for the overexpression of *fucO* and *aldA* genes

Primer name	Sequence (5'-3')
pCA24N_frame_1	tttagcttccttagctcctga
pCA24N_frame_2	tttttttaaggcagttattggtgccc
ASKA-Gibson_Kan_Fw	caggagctaaggaagctaaaaatatgtatccgctcatgag
ASKA-Gibson_Kan_Rev	gggcaccaataactgccttaaaaaaaatgaagttttgacggtatcg
ASKA-S2	attaagcttggctgcaggtc
aldA-1	ggaagcgttgcctgctattg

Supplementary Methods

Metabolic network simulations

Reconstruction of the universal reaction set

To study the potential adaptive value of adding new reactions to the *E. coli* metabolic network, we compiled a dataset of metabolic reactions reported from species across the three kingdoms of life ("universal reaction set") and absent from *E. coli*. First, we mapped the metabolites of the manually curated *Escherichia coli* genome-scale metabolic model [6] to the Model SEED database [7] (and http://blog.theseed.org/model_seed/), a comprehensive resource for automatically generated genome-scale metabolic network reconstructions. Because Model SEED does not contain the most recent version (iJO1366 [8]) of the *E. coli* network reconstruction, we used an earlier version (iAF1260 [6]) that is widely utilized and has been extensively tested [9]. As a second step, we added all mass-balanced biochemical reactions from the Model SEED database to the *E. coli* model. From this draft network, we removed duplicate reactions. Next, we removed 'perpetuum mobile' cycles as follows.

Due to incorrect annotations or to the merging of incompatible metabolic systems during the construction of the universal reaction set, we observed 'perpetuum mobile' futile cycles, i.e., flux distributions capable of producing energy without consuming any nutrients. To remove such thermodynamically impossible cycles, we formulated an algorithm based on a mixed integer linear problem (MILP) as follows. First, we added 15 energy dissipation reactions (Supplementary Table 5). These irreversible reactions split high energy currency metabolites into their lower energy counterparts or embody energy-demanding transformation or transport of metabolites. E.g. one of these reactions is used in multiple metabolic models to simulate the ATP maintenance energy [6]:

$ATP + H_2O \rightarrow ADP + P_i + H^+$

The presence of a 'perpetuum mobile' cycle is indicated by a positive flux through one of these reactions when all nutrient uptakes are inhibited in the model. By now minimizing the number of active reactions while constraining metabolite uptake to zero and requiring that at least one energy dissipation reaction carries a positive flux, the result will be one minimal 'perpetuum mobile' futile cycle. We thus iteratively solve the following problem:

$$\text{minimize} \sum b_i$$

$$\text{s.t.:}$$

$$Sv = 0$$

$$l_i \leq v_i \leq u_i$$

$$(b_i = 0) \Rightarrow (v_i = 0)$$

$$\sum_{j \in D} v_j > threshold$$

$$v \in \mathbb{R}^n$$

$$b \in \{0,1\}^n$$

$$D \subset \{1, \dots, n\}$$

$$\forall\, i \in \{1, \dots, n\}$$

Where *S (m x n)* is the stoichiometric matrix of the metabolic network, *v (n x 1)* is a vector of real numbers representing the *n* reaction fluxes, *b (n x 1)* is a binary vector consisting only of zeros and ones, and vectors of the upper and lower reaction bounds are *u* and *l*, respectively. Each flux value v_i is a binary variable b_i assigned that implies $v_i = 0$ if $b_i = 0$. Set *D* contains the indices of the newly added energy dissipation reactions. The threshold was set to 10 for numerical reasons.

For each reaction in the identified cycle, we determined the minimal phylogenetic distance of its occurrence relative to *E. coli*. We then impaired the cycle by removing one reaction. If reactions without any phylogenetic assignments were part of the cycle, we removed one of those; otherwise we removed a reaction for which the phylogenetic distance to *E. coli* was maximal. We

then started the next iteration by checking if the reduced network could still produce energy without any nutrient uptake. We stopped the iterations when the network was no longer producing energy in the absence of nutrients. Finally, we removed unconditionally blocked reactions (i.e. those unable to carry a flux under any condition). The resulting curated universal reaction network contains 4949 metabolic reactions and 444 nutrient uptake reactions, of which 2566 and 159 are not present in the *E. coli* network, respectively. The universal network is available as a computational SBML model (Supplementary Data 4).

Supplementary References

1. Sarkar S, Ma WT, Sandri GH. On fluctuation analysis: a new, simple and efficient method for computing the expected number of mutants. *Genetica* **85**, 173-179 (1992).

2. Hall BM, Ma CX, Liang P, Singh KK. Fluctuation analysis CalculatOR: a web tool for the determination of mutation rate using Luria-Delbruck fluctuation analysis. *Bioinformatics* **25**, 1564-1565 (2009).

3. Sabarly V, *et al.* The decoupling between genetic structure and metabolic phenotypes in Escherichia coli leads to continuous phenotypic diversity. *J Evol Biol* **24**, 1559-1571 (2011).

4. Hansen LH, Knudsen S, Sorensen SJ. The effect of the lacY gene on the induction of IPTG inducible promoters, studied in Escherichia coli and Pseudomonas fluorescens. *Curr Microbiol* **36**, 341-347 (1998).

5. Blattner FR, *et al.* The complete genome sequence of Escherichia coli K-12. *Science* **277**, 1453-1462 (1997).

6. Feist AM, *et al.* A genome-scale metabolic reconstruction for Escherichia coli K-12 MG1655 that accounts for 1260 ORFs and thermodynamic information. *Mol Syst Biol* **3**, 121 (2007).

7. Henry CS, DeJongh M, Best AA, Frybarger PM, Linsay B, Stevens RL. High-throughput generation, optimization and analysis of genome-scale metabolic models. *Nat Biotechnol* **28**, 977-982 (2010).

8. Orth JD, *et al.* A comprehensive genome-scale reconstruction of Escherichia coli metabolism--2011. *Mol Syst Biol* **7**, 535 (2011).

9. McCloskey D, Palsson BO, Feist AM. Basic and applied uses of genome-scale metabolic network reconstructions of Escherichia coli. *Mol Syst Biol* **9**, 661 (2013).
